# Supplementary figures and images for: Core training elicits greater improvements than flexibility training in jumping lotus kick performance and physical attributes of Tai Chi athletes: A randomized controlled trial
Source: PLoS One. 2025 Dec 23;20(12):e0335431. doi: 10.1371/journal.pone.0335431 (PMC12725596; doi:10.1371/journal.pone.0335431)

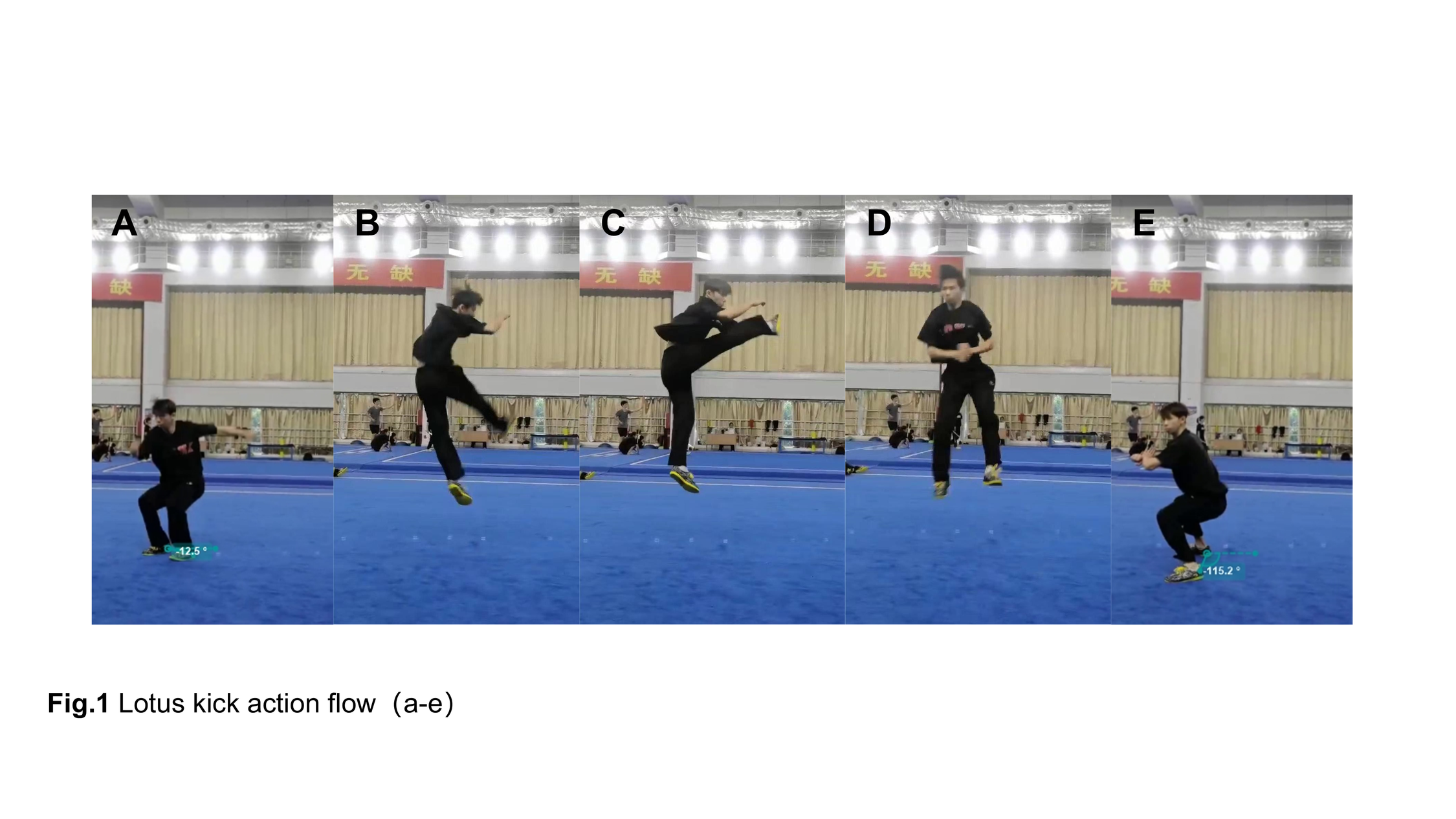

Supplement: S1 Fig — The figure depicts the progression through each phase of the Jumping Lotus Kick, highlighting the initial rotational angle (−12.5°) and final rotational angle (−115.2°) as quantified using Kinovea software. (TIF) [file pone.0335431.s001.tif]

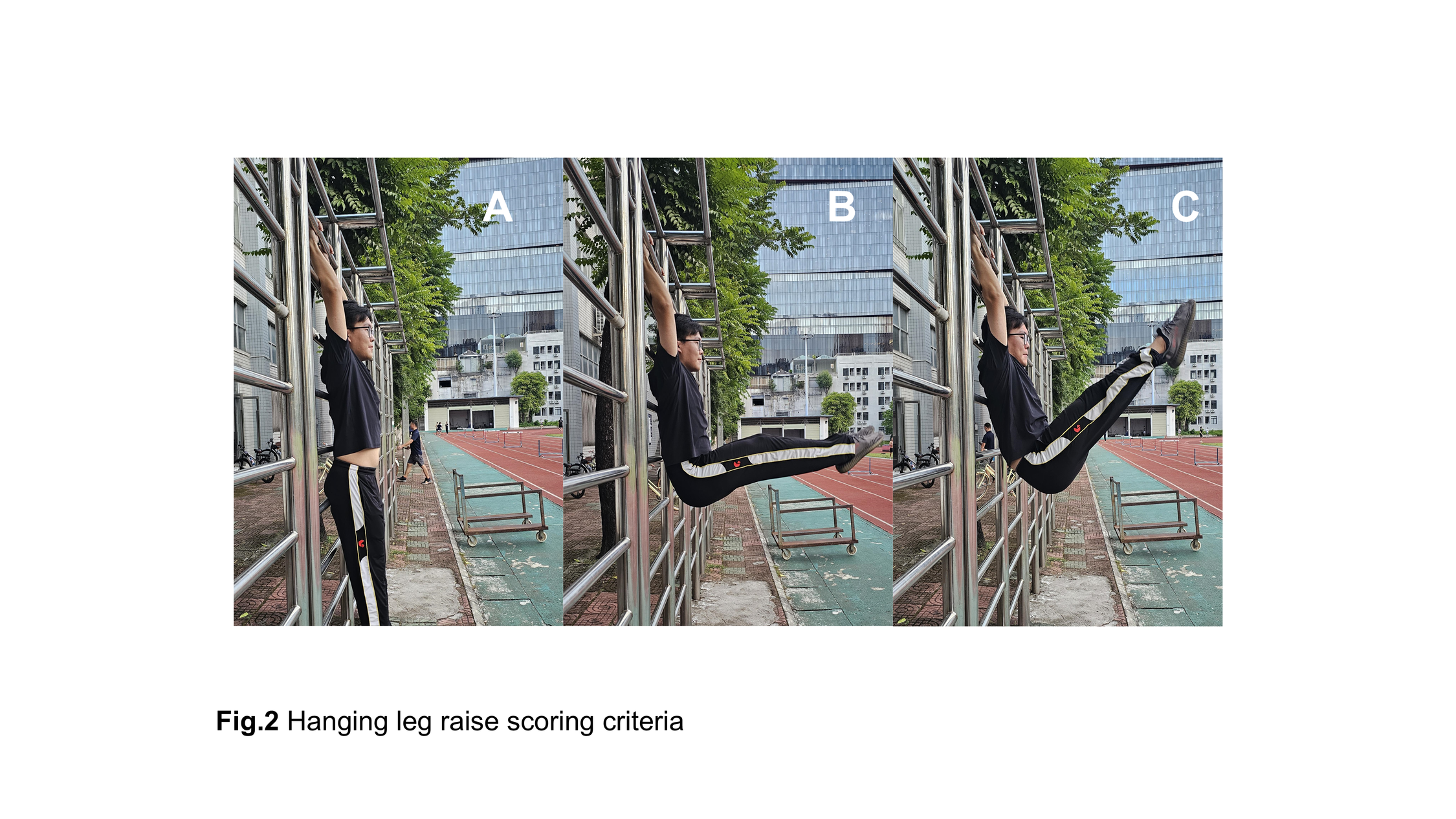

Supplement: S2 Fig — (A)Starting position, (B) Intermediate horizontal and (C) Final raised position. (TIF) [file pone.0335431.s002.tif]

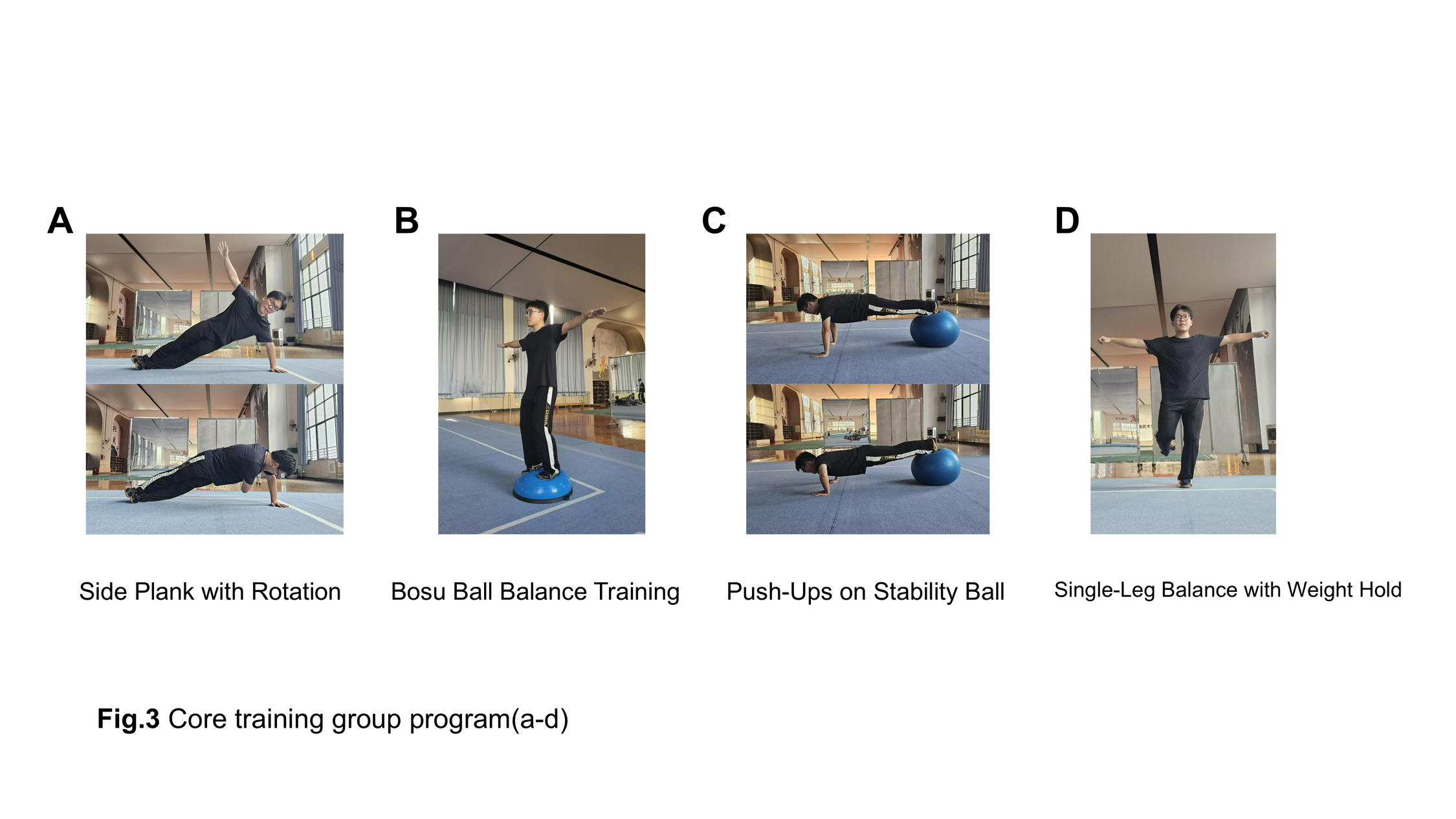

Supplement: S3 Fig — (A) Side plank with rotation, (B) Bosu ball balance, (C) Stability ball push-ups and (D) Single-leg balance with weight. (TIF) [file pone.0335431.s003.tif]

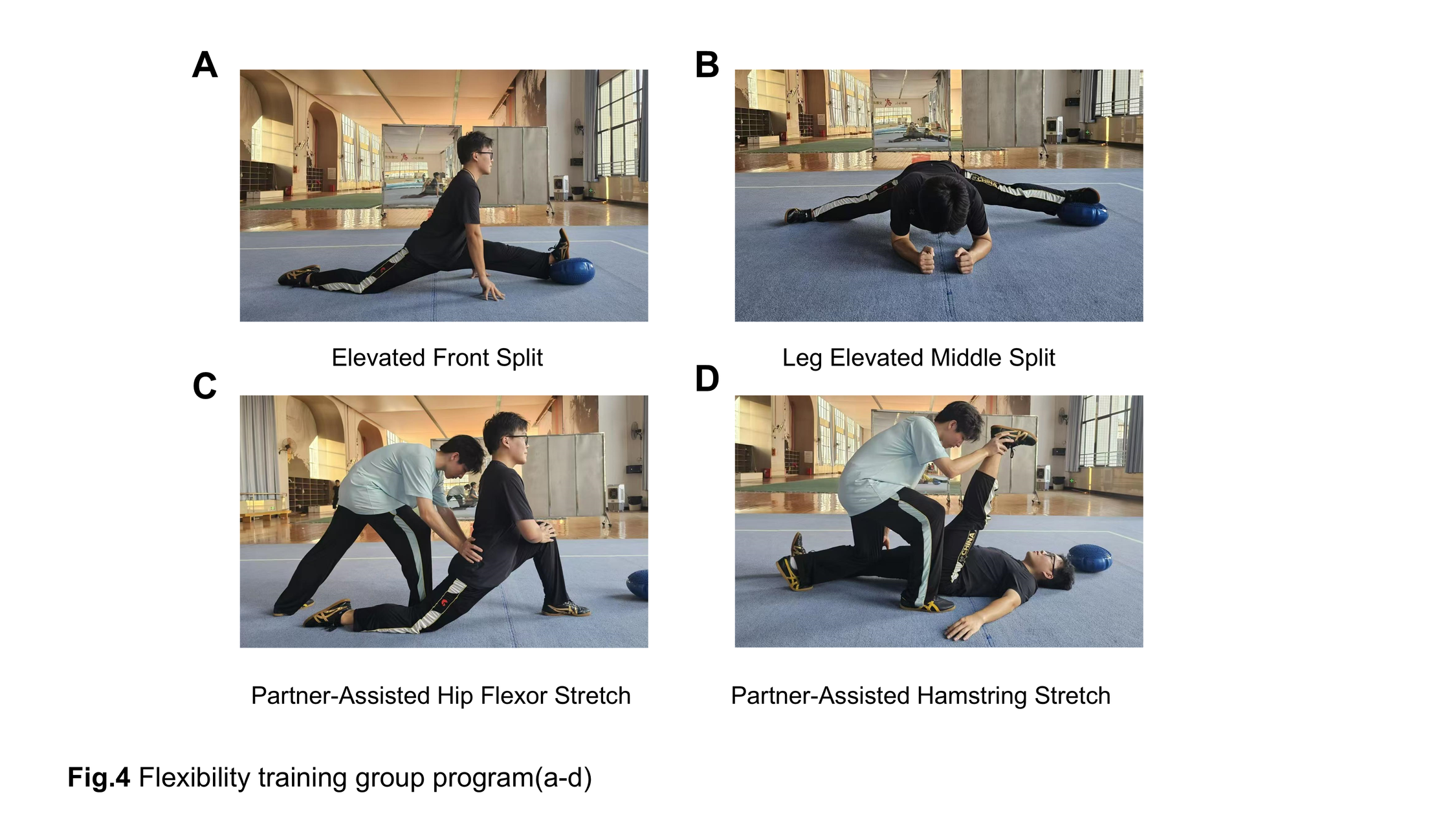

Supplement: S4 Fig — (A) Elevated front split, (B) Leg elevated middle split, (C) Partner-Assisted Hip Flexor Stretch and (D) Partner-Assisted Hamstring Stretch. (TIF) [file pone.0335431.s004.tif]
