# Supplementary material for: Core training elicits greater improvements than flexibility training in jumping lotus kick performance and physical attributes of Tai Chi athletes: A randomized controlled trial
Source: PLoS One. 2025 Dec 23;20(12):e0335431. doi: 10.1371/journal.pone.0335431 (PMC12725596; doi:10.1371/journal.pone.0335431)
Supplement: S1 Appendix — This appendix provides a detailed breakdown of the core training exercises, sets, repetitions per side, rest intervals, and progression framework across an 8-week regimen aimed at systematically improving core stability and dynamic balance. (DOCX) [file pone.0335431.s005.docx]

**S1 Table. Core training session details**

| **Exercise** | **Sets** | **Repetitions/Duration per side** | **Rest Interval** | **Progression** |
| --- | --- | --- | --- | --- |
| Side Plank with Rotation | 3-4 | 10-15 repetitions | 30-60 seconds | Weeks 1-2: 3x10 reps; Weeks 3-4: 3x12 reps; Weeks 5-6: 4x14 reps; Weeks 7-8: 4x15 reps |
| Bosu Ball Balance Training | 3-4 | 30-60 seconds | 30-60 seconds | Weeks 1-2: 3x30 seconds; Weeks 3-4: 3x40 seconds; Weeks 5-6: 4x50 seconds; Weeks 7-8: 4x60 seconds |
| Push-Ups on Stability Ball | 3-4 | 10-15 repetitions | 30-60 seconds | Weeks 1-2: 3x10 reps; Weeks 3-4: 3x12 reps; Weeks 5-6: 4x14 reps; Weeks 7-8: 4x15 reps |
| Single-Leg Balance with Weight Hold | 3-4 | 60 seconds | 30-60 seconds | Weeks 1-2: 3x60 seconds, zero weight; Weeks 3-4: 3x60 seconds, lighter weight; Weeks 5-6: 4x60 seconds, moderate weight; Weeks 7-8: 4x60 seconds, increased weight |

### ****Core Training Exercises Description****

**Side Plank with Rotation**

The participant begins in a side plank position, with the supporting arm fully extended and aligned directly under the shoulder, ensuring optimal joint positioning to avoid unnecessary strain. The contralateral arm is raised overhead, and the body is held in a straight line from head to feet, engaging the entire core and lower body to maintain stability. The movement involves a controlled rotation of the torso, bringing the raised arm toward the floor while maintaining stability in the lower body. This exercise primarily engages the obliques, transversus abdominis, and multifidus, enhancing core stability and shoulder mobility. (1,2)The body’s alignment is maintained throughout the rotation to prevent compensatory movements that may reduce the exercise's efficacy or increase injury risk.

**Bosu Ball Balance Training**

The participant stands on the flat surface of a Bosu ball with feet shoulder-width apart and arms extended horizontally for balance. This exercise increases the demands on core stabilizers and lower limb musculature, particularly targeting the ankle, knee, and hip stabilizers to maintain equilibrium.(3) It enhances proprioceptive awareness and neuromuscular coordination, contributing to improved dynamic balance. (4–6)The instability of the Bosu ball activates the deep stabilizing muscles of the trunk (e.g., transversus abdominis, lumbar multifidus) while preventing excessive movement in the lower body. Progression in this exercise can involve longer hold durations or dynamic movements such as shifting weight between feet, thereby increasing the challenge for core stabilizers.

**Push-Ups on Stability Ball**

The participant performs push-ups with feet placed on a stability ball, creating an unstable support surface that significantly increases the activation of core stabilizers. This exercise predominantly targets the pectoralis major, deltoid muscles, triceps brachii, and rectus abdominis. The instability introduced by the ball necessitates continuous engagement of deep core musculature, particularly the transversus abdominis, multifidus, and the erector spinae, to maintain postural alignment and control.(7)The movement should be performed slowly and in a controlled manner, ensuring that the spine is in a neutral position to avoid excessive rolling of the ball, avoid deviations in the movement that can affect the exercise effect, and reduce the risk of injury.

**Single-Leg Balance with Weight Hold**

In this exercise, the participant stands on one leg while holding a weight at chest height, with arms extended either forward or laterally. The contralateral leg is lifted and held in a flexed position. This exercise primarily activates lower limb stabilizers (gluteus medius, quadriceps, and soleus) and engages core muscles (transversus abdominis and multifidus) to maintain postural stability. The unilateral stance enhances proprioceptive feedback and neuromuscular coordination, improving dynamic balance and reducing injury risk.(8)

The weight progression follows a structured increase every two weeks: Weeks 1-2 involve no external weight, focusing on technique; Weeks 3-4 introduce 2 kg per hand, Weeks 5-6 increase to 3 kg per hand, and Weeks 7-8 raise the load to 5 kg per hand. This gradual progression allows for strength and stability development without compromising form or causing compensatory movements.(8)

### ****References****

1. Escamilla RF, Lewis C, Pecson A, Imamura R, Andrews JR. Muscle Activation Among Supine, Prone, and Side Position Exercises With and Without a Swiss Ball. Sports Health: A Multidisciplinary Approach. 2016 Jul;8(4):372–9.

2. Imai A, Kaneoka K, Okubo Y, Shiina I, Tatsumura M, Izumi S, et al. Trunk Muscle Activity During Lumbar Stabilization Exercises on Both a Stable and Unstable Surface. Journal of Orthopaedic & Sports Physical Therapy. 2010 Jun;40(6):369–75.

3. Cuğ M, Duncan A, Wikstrom E. Comparative effects of different balance-training–progression styles on postural control and ankle force production: a randomized controlled trial. Journal of athletic training. 2016;51(2):101–10.

4. Cerrah AO, Bayram İ, Yıldızer G, Uğurlu O, Şimşek D, Ertan H. Effects of functional balance training on static and dynamic balance performance of adolescent soccer players. International Journal of Sport Exercise and Training Sciences-IJSETS. 2016;2(2):73–81.

5. Kümmel J, Kramer A, Giboin LS, Gruber M. Specificity of Balance Training in Healthy Individuals: A Systematic Review and Meta-Analysis. Sports Med. 2016 Sep;46(9):1261–71.

6. Oliver GD, Di Brezzo R. Functional balance training in collegiate women athletes. The Journal of Strength & Conditioning Research. 2009;23(7):2124–9.

7. Dyrek AJ. Agonist and Stabilizer Muscle Activity during a Push Up on Unstable Surfaces. [cited 2024 Oct 15]; Available from: https://digitalscholarship.unlv.edu/thesesdissertations/1083

8. Rasool J, George K. The impact of single-leg dynamic balance training on dynamic stability. Physical therapy in sport. 2007;8(4):177–84.
