# Supplementary material for: Core training elicits greater improvements than flexibility training in jumping lotus kick performance and physical attributes of Tai Chi athletes: A randomized controlled trial
Source: PLoS One. 2025 Dec 23;20(12):e0335431. doi: 10.1371/journal.pone.0335431 (PMC12725596; doi:10.1371/journal.pone.0335431)
Supplement: S2 Appendix — This appendix provides a detailed breakdown of the exercises, sets, duration per side, rest intervals, and progression framework across an 8-week flexibility training regimen aimed at systematically improving range of motion and dynamic flexibility. (DOCX) [file pone.0335431.s006.docx]

**S2 Table. flexibility training session details**

| **Exercise** | **Sets** | **Duration/Repetitions per side** | **Rest Interval** | **Progression** |
| --- | --- | --- | --- | --- |
| Elevated Front Split | 2-3 | 25-40 seconds | 45 seconds | Weeks 1-2: 2x25s; Weeks 3-4: 2x30s; Weeks 5-6: 3x35s; Weeks 7-8: 3x40s with increased intensity |
| Leg Elevated Middle Split | 2-3 | 25-40 seconds | 45 seconds | Weeks 1-2: 2x25s; Weeks 3-4: 2x30s; Weeks 5-6: 3x35s; Weeks 7-8: 3x40s with increased intensity |
| Partner-Assisted Hip Flexor Stretch | 2-3 | 25-40 seconds | 60 seconds | Weeks 1-2: 2x25s; Weeks 3-4: 2x30s; Weeks 5-6: 3x35s; Weeks 7-8: 3x40s with increased intensity |
| Partner-Assisted Hamstring Stretch | 2-3 | 25-40 seconds | 60 seconds | Weeks 1-2: 2x25s; Weeks 3-4: 2x30s; Weeks 5-6: 3x35s; Weeks 7-8: 3x40s with increased intensity |

**Flexibility Training Exercises Description**

**Elevated Front Split**

**This advanced flexibility exercise targets the hip flexors and hamstrings. The rear leg is elevated on a stable surface, which increases stretch intensity by placing greater tension on these muscles, while the front leg remains extended on the ground. The athlete’s hands are placed on the floor for support and alignment.**(1) **Intensity is progressively increased every two weeks by extending the range of motion and hold duration. By Weeks 7-8, intensity is further increased through deeper stretch angles and active engagement of the quadriceps and gluteal muscles, promoting both control and deeper flexibility.**(2) **This exercise is essential for improving hip mobility, crucial for performing high kicks in martial arts, and for reducing the risk of injury by enhancing muscle elasticity and joint range of motion.**

**Leg Elevated Middle Split**

**In this exercise, the athlete performs a middle split with both legs elevated on stable platforms or cushions. The torso is gradually lowered toward the floor, increasing tension on the adductor and hamstring muscles. Intensity is increased biweekly by lowering the chest further or extending the hold time, thereby enhancing flexibility. By Weeks 7-8, the athlete is encouraged to actively engage the quadriceps and gluteal muscles, deepening the stretch while maintaining proper alignment. This progressive stretching approach improves range of motion for lateral kicking techniques and helps prevent muscle strain during high-intensity movements.**

**Partner-Assisted Hip Flexor Stretch**

**The athlete assumes a deep lunge position, with one knee resting on the floor and the other leg bent at a 90-degree angle. A partner applies controlled pressure to the hips and lower back to ensure alignment and to deepen the stretch. Every two weeks, intensity is increased by slightly increasing the applied pressure or encouraging the athlete to engage the hip flexors actively. By Weeks 7-8, the partner further assists in increasing the range of motion, while ensuring the athlete remains within a safe and comfortable limit. This exercise specifically targets the hip flexors and quadriceps, which are essential for flexibility in explosive leg movements like kicking. Partner-assisted stretching ensures a gradual, controlled increase in flexibility, reducing the risk of overstretching.**

**Partner-Assisted Hamstring Stretch**

**In this passive stretch, the athlete lies supine while a partner lifts one leg toward the torso, keeping the opposite leg extended on the floor**. (3,4)**The stretch focuses on the hamstring muscles, crucial for high-velocity kicks. Every two weeks, intensity is increased by raising the leg higher or extending the hold time.** (4)**In Weeks 7-8, the intensity is further amplified by incorporating longer hold times or slightly increasing the elevation of the leg, with careful partner control to prevent overstretching. This method allows for a gradual improvement in hamstring flexibility, ensuring safety and minimizing injury risks through controlled and incremental range of motion increases.(3)**

**References**

1. Sands WA, McNeal JR, Stone MH, Kimmel WL, Gregory Haff G, Jemni M. The effect of vibration on active and passive range of motion in elite female synchronized swimmers. European Journal of Sport Science. 2008 Jul;8(4):217–23.

2. Freitas SR, Mil-Homens P. Effect of 8-week high-intensity stretching training on biceps femoris architecture. The Journal of Strength & Conditioning Research. 2015;29(6):1737–40.

3. McAtee RE. Facilitated stretching [Internet]. Human kinetics; 2013 [cited 2024 Oct 15]. Available from: https://books.google.com/books?hl=zh-CN&lr=&id=GvB6DwAAQBAJ&oi=fnd&pg=PR1&dq=Partner-Assisted+Stretching%EF%BC%8CHip+Flexor+Flexibility%EF%BC%8CQuadriceps+Stretching%EF%BC%8CDeep+Lunge+Stretch&ots=LlZh1ps-1O&sig=hSCfY9zw_LHjgb3lA0G7rQeTwPE

4. Behm D. The science and physiology of flexibility and stretching: implications and applications in sport performance and health [Internet]. Taylor & Francis; 2024 [cited 2024 Oct 15]. Available from: https://books.google.com/books?hl=zh-CN&lr=&id=MUgaEQAAQBAJ&oi=fnd&pg=PT13&dq=Progressive+Stretching,+Flexibility+Training+for+High+Kicks,+Martial+Arts+Flexibility&ots=GT8Mxy26C0&sig=5Y-ZYulXCr0lk0D3AgqMGFOywk4
