# Supplementary material for: Core training elicits greater improvements than flexibility training in jumping lotus kick performance and physical attributes of Tai Chi athletes: A randomized controlled trial
Source: PLoS One. 2025 Dec 23;20(12):e0335431. doi: 10.1371/journal.pone.0335431 (PMC12725596; doi:10.1371/journal.pone.0335431)
Supplement: Attachment — Submitted filename: Review PONE-D-24-44478.docx [file pone.0335431.s007.docx]

Review of the manuscript PONE-D-24-44478, entitled " Core Training Elicits Greater Improvements Than Flexibility Training in Jumping Lotus Kick Performance and Physical Attributes of Tai Chi Athletes: A Randomized

Controlled Trial"

The article presents a study aimed to compare the impacts of an eight-week core stability regimen versus flexibility training on JL Kick proficiency and related physical attributes in Tai Chi athletes.

This study highlights the superior effectiveness of core stability training over flexibility training in improving Jumping Lotus Kick performance and related physical attributes among male Wushu athletes. By emphasizing the importance of core strength, balance, and reactive strength, the findings advocate for a more comprehensive approach to martial arts conditioning, challenging traditional flexibility-focused training models. By incorporating core stability training into conditioning regimens, martial artists and athletes in other dynamic sports may unlock greater potential for skill development and competitive success, ultimately reshaping the conditioning strategies in high-performance disciplines.

The study presents the results of original research. The introduction section is well presented although references 14, 17, 42, 43 had to be edited as the title is in capital letters. Maybe could be added some extra recent investigation about the effects of core training on flexibility in other sports which requires also high bouts of dynamic stretching.

Study design, participants, training protocol, experiments, statistics, and other analyses are performed to a high technical standard and are described in sufficient detail. Conclusions are presented in an appropriate fashion. The article is presented in an intelligible fashion and is written in standard English.

In my opinion, specific minor changes must to be amended and considered before acceptance.

Comment #1: lines 49-50.

The authors state “While static stretching traditionally improves range of motion and mitigates injury risk, recent findings highlight the need for dynamic flexibility, aiding in neuromuscular control during explosive movements”, nevertheless their purpose for the FTG is based in static stretching. Please justify why did you choose static stretching if you already knew that is less efficient to improve explosive sports performance as the Jumping Lotus Kick?

Comment #2: Lines 130-139

The authors state: “The main exercises included three core stability exercises performed for 3 sets of 12-15 repetitions” and “A rest period of 1-2 minutes was provided between sets to support muscle recovery”. Normally, core stability programs aimed to improve the resistance of the core muscles. The purposed sets and repetitions are into the range of sets and repetitions included in resistance adaptations, nevertheless, the resting periods should be lower than 60” to avoid total recovery of core muscles and thus improve their adaptation to core stability. Despite of this, there were positive adaptations to this setup. Maybe in future research the authors should consider to purpose more challenging resting periods.

Comment #3: lines 143-144

The authors state:” The main stretching routine consisted of 4-5 stretches per session, held for 30-40 seconds each”. I miss some information about the amount of stretching tension supported by the participants as 30-40 sec is reported to be not enough to produce any effect on hamstrings elongation. Why don’t you apply longer stretching times? As is reported that stretching effects are better when the exposure times are longer, more than doing more than one set of shorter stretches.

Comment #4: 147-149

The authors state “Intensity was progressively increased bi-weekly by extending hold times or using assisted techniques, as needed. Participants rested for 30 seconds between stretches to prevent fatigue”. When did you applied assisted techniques or increasing the time? Justify the decision.

Moreover in lines

Comment #5: lines 155-157

The authors state: “The core group progressed through increased

repetitions or resistance, while the flexibility group extended stretch durations”. Could you justify by previous studies the chosen method to increase the time exposure in stretches? Was Borg 0-10 scale also be used for stretches?

Did you consider any method to assess athletes’ perception of stretch intensity?

Comment #6: line 270

Table format should be improved.

Comment #7: line 283

Table format should be improved.

Comment #8: line 313

Table format should be improved.

Comment #9: line 326

Table format should be improved.

Comment #10: line 337-341

The authors state “findings contribute to a growing discourse challenging traditional flexibility-centered conditioning approaches in martial arts and position core stability as an essential component for technical mastery in disciplines emphasizing control, balance, and precision.” You should express the limitations on doing static stretches, as is highly demonstrated that static stretches reduce power and jump performance, so needed in martial arts.

Coment #11: lines 372-374

The authors state: “This improvement suggests that core stability training directly enhances proprioceptive control, a critical factor in executing movements that require balanced rotational shifts”. Not all core stability exercises have this effect. Proprioception highly depends on imagery, focusing attention and reduce other afferent inputs that can negative interfere in the proprioceptive signal entry. Be careful on comparing proprioception with balance performance, as doing balance exercise not always improve the proprioceptive sense.
