# Supplementary material for: Core training elicits greater improvements than flexibility training in jumping lotus kick performance and physical attributes of Tai Chi athletes: A randomized controlled trial
Source: PLoS One. 2025 Dec 23;20(12):e0335431. doi: 10.1371/journal.pone.0335431 (PMC12725596; doi:10.1371/journal.pone.0335431)
Supplement: Attachment — Submitted filename: Response to Reviewers.docx [file pone.0335431.s008.docx]

**Response to Reviewer Comments**

**Manuscript Title**: Core Training Elicits Greater Improvements Than Flexibility Training in Jumping Lotus Kick Performance and Physical Attributes of Tai Chi Athletes: A Randomized Controlled Trial

**Manuscript ID**: PONE-D-24-44478

We would like to thank the Academic Editor and the reviewer for their constructive comments and valuable suggestions. We have revised our manuscript accordingly and provide detailed responses to each point below. All modifications have been marked in the revised manuscript with track changes.

**Reviewer #1 Comments:**

**Comment 1:**

Lines 49–50. The authors state: “While static stretching traditionally improves range of motion and mitigates injury risk, recent findings highlight the need for dynamic flexibility, aiding in neuromuscular control during explosive movements”, nevertheless their purpose for the FTG is based in static stretching. Please justify why did you choose static stretching if you already knew that is less efficient to improve explosive sports performance as the Jumping Lotus Kick?

**Response:**

We sincerely appreciate the reviewer's insightful observation regarding the potential discrepancy between our acknowledgment in the introduction of dynamic flexibility's benefits for neuromuscular control in explosive movements and the static-dominant protocol in the flexibility training group (FTG). We acknowledge that this rationale requires clearer articulation to align with the cited literature.

The FTG was designed to reflect traditional Tai Chi and Wushu practices, where static stretching post-training enhances range of motion (ROM) and reduces injury risk, as demonstrated by Bandy et al. (1998) in hamstring flexibility studies. Dynamic elements, such as kicks, were integrated into routine sessions, allowing FTG to leverage warmed muscles and avoid acute decrements (Behm et al., 2016). This enabled a valid comparator, yielding ROM gains (Sit and Reach, ES=1.44) without hindering Jumping Lotus Kick performance.

To enhance transparency, we propose revising the introduction (Lines 48–50) to: "While static stretching improves ROM, reduces injury, and prevails in post-training martial arts, recent evidence favors dynamic flexibility for explosive neuromuscular control(13,14) " .

In methods (Lines 142–144) add: "To mirror conventional martial arts protocols, the FTG focused on static stretching post-Tai Chi, complementing the dynamic kicks inherent in the standard sessions shared across groups".

**Comment 2:**

Lines 130–139. The authors state: “The main exercises included three core stability exercises performed for 3 sets of 12-15 repetitions” and “A rest period of 1-2 minutes was provided between sets to support muscle recovery”. Normally, core stability programs aimed to improve the resistance of the core muscles. The purposed sets and repetitions are into the range of sets and repetitions included in resistance adaptations, nevertheless, the resting periods should be lower than 60” to avoid total recovery of core muscles and thus improve their adaptation to core stability. Maybe in future research the authors should consider to purpose more challenging resting periods.

**Response:**

We thank the reviewer for their insightful comment on the 1-2 minute rest intervals in the core training group (CTG) protocol. These intervals may favor strength adaptations over endurance. This could be less optimal for Tai Chi's sustained rotational demands. Shorter intervals (<60 seconds) could induce greater metabolic stress. The chosen intervals aimed to promote ATP-CP resynthesis. This ensures high-quality repetitions vital for Tai Chi's postural precision and control (Willardson, 2006). This approach led to notable gains in core strength (ES=1.39) and performance (ES=2.16 vs. control). It aligns with findings that 1-3 minute rests enhance strength in multi-set resistance protocols (de Salles et al., 2009). Still, we acknowledge the potential endurance shortfall.

To enhance clarity, we will revise methods (Lines 139–141): "A rest period of 1-2 minutes was provided between sets to support muscle recovery and balance strength-endurance adaptations for Tai Chi demands (20,21)."

In discussion: Combines Comments 2 and 5 into a single, concise sentence, replacing the original biomechanical sentence and rest interval insertion, aligning with paragraph's future research focus (Lines 425–427)："Future research might employ biomechanical tools and RPE scales to enhance monitoring of FTG stretch intensity and CTG endurance, ensuring protocol-specific data capture (24,29)."

**Comment 3:**

(Lines 143-144): The authors state: “The main stretching routine consisted of 4-5 stretches per session, held for 30-40 seconds each”. I miss some information about the amount of stretching tension supported by the participants as 30-40 sec is reported to be not enough to produce any effect on hamstrings elongation. Why don’t you apply longer stretching times? As is reported that stretching effects are better when the exposure times are longer, more than doing more than one set of shorter stretches.

**Response:**

We thank the reviewer for noting the hold durations of 30–40 seconds in the flexibility training group (FTG) protocol, including concerns about sufficiency for elongation and omitted tension details. We recognize these points merit explicit clarification to bolster reproducibility.

Durations were selected for athlete tolerability, emphasizing cumulative exposure (2–3 minutes per muscle group across 4–5 stretches), aligned with meta-analyses showing ROM improvements from accumulated volumes (Page, 2012). Tension was maintained at moderate discomfort, a standard safety cue, though not formally quantified.

We will revise methods (Lines 145–147) to: "The routine included 4-5 stretches per session, held 30–40 seconds at moderate discomfort for tolerability and 2–3 minutes cumulative exposure per group (22). "

**Comment 4:**

Lines 147–149. The authors state: “Intensity was progressively increased bi-weekly by extending hold times or using assisted techniques, as needed. Participants rested for 30 seconds between stretches to prevent fatigue.” When did you apply assisted techniques or increased hold times? Justify the decision.

**Response:**

We appreciate the reviewer's call for details on progression timing and criteria in the flexibility training group (FTG) protocol. We admit the original text lacked sufficient specificity, impacting perceived rigor.

Progression was bi-weekly post-initial adaptation, decided via participant tolerance and coach observations, per progressive overload guidelines (American College of Sports Medicine, 2009). This ensured safe escalation, such as hold extensions or assisted techniques.

We will revise methods (Lines 150–153) to: "Progression occurred bi-weekly after the initial adaptation phase, with decisions guided by participant tolerance (e.g., absence of excessive fatigue) and coach observations per overload guidelines (22,23). "

**Comment 5:**

Lines 155–157. The authors state: “The core group progressed through increased repetitions or resistance, while the flexibility group extended stretch durations”. Could you justify by previous studies the chosen method to increase the time exposure in stretches? Was Borg 0–10 scale also used for stretches? Did you consider any method to assess athletes’ perception of stretch intensity?

**Response:**

We thank the reviewer for querying the progression method, Borg RPE application, and perceived intensity assessments in the flexibility training group (FTG) protocol. We concede these details were under-elaborated, constituting a methodological limitation.

Bi-weekly duration increases followed progressive overload, supported by evidence of ROM gains from gradual volume escalation over ≥2 weeks (Konrad et al., 2024). RPE was not applied to stretches, relying on informal cues, unlike core sessions.

We will revise methods (Lines 159–163) to: "with the core group increasing repetitions or resistance and the flexibility group extending stretch durations—supported by evidence of ROM gains from gradual volume escalations over ≥2 weeks (24). Session intensity was monitored via the Borg 0-10 RPE scale for the CTG only, recorded post-session for subjective workload (25) " .
In discussion: Combines Comments 2 and 5 into a single, concise sentence, replacing the original biomechanical sentence and rest interval insertion, aligning with paragraph's future research focus (Lines 425–427)："Future research might employ biomechanical tools and RPE scales to enhance monitoring of FTG stretch intensity and CTG endurance, ensuring protocol-specific data capture (24,49)."

**Comment 6–9:**

Table format should be improved (Tables 2–5).

**Response:**

We appreciate the reviewer’s suggestion. We have reformatted all tables according to PLOS ONE style guidelines, placing table titles above the tables, standardizing unit presentation, and aligning all values for improved clarity and readability.

**Comment 10:**

Lines 337–341. You should express the limitations on doing static stretches, as is highly demonstrated that static stretches reduce power and jump performance, so needed in martial arts.

**Response:**

We thank the reviewer for emphasizing the need to address limitations of static stretching in the discussion, particularly its acute reductions in power and jump performance, essential for martial arts. We acknowledge this as a valid point, as our flexibility training group (FTG) featured static-dominant elements, and omitting these effects could undervalue explanations for limited explosive outcomes.

Static stretching can impair immediate power and reactive strength, with meta-analyses indicating moderate decrements in jump height and force post-holds exceeding 60 seconds (Behm et al., 2016). Our FTG, while yielding range of motion gains (Sit and Reach, ES=1.44), showed minimal Rebound Jump Index or Jumping Lotus Kick improvements versus core training, likely influenced by these neuromuscular inhibitions (Warneke & Lohmann, 2024). This underscores stretching timing in dynamic disciplines, where post-training static approaches mitigate some risks but retain performance trade-offs.

To integrate this, we will revise the discussion (Lines 403–405) to add: " This is particularly evident given static stretching’s acute reductions in power and jump performance, which likely limited FTG’s explosive outcomes, underscoring the need for dynamic approaches (45,46)."

**Comment 11:**

Lines 372–374. Not all core stability exercises have this effect. Proprioception highly depends on imagery, focusing attention and reduction of competing afferent inputs. Be careful on comparing proprioception with balance performance.

**Response:**

We appreciate the reviewer's nuanced point on core stability exercises' effects on proprioception, underscoring its reliance on imagery, attention, and afferent modulation. We concede this risks conflating proprioception with balance, a distinction essential for accurate interpretation.

Proprioception involves sensory integration, while balance is a functional output; core training may enhance the latter without uniformly affecting the former, as Hrysomallis (2011) notes in reviewing balance's role in athletic performance, where core interventions improve stability via neuromuscular pathways but vary in proprioceptive impact.

To refine, we will revise discussion (Lines 377–379): "Core stability training enhances neuromuscular balance control, a critical factor for rotational shifts, though often conflated with proprioception, which involves sensory integration (38)."

**Once again, we thank the reviewer for their thorough evaluation and constructive comments, which have greatly improved the quality and clarity of our manuscript.**
